# Molecular apocrine breast cancers are aggressive estrogen receptor negative tumors overexpressing either HER2 or GCDFP15

**DOI:** 10.1186/bcr3421

**Published:** 2013-05-11

**Authors:** Jacqueline Lehmann-Che, Anne-Sophie Hamy, Raphaël Porcher, Marc Barritault, Fatiha Bouhidel, Hanadi Habuellelah, Solenne Leman-Detours, Anne de Roquancourt, Laurence Cahen-Doidy, Edwige Bourstyn, Patricia de Cremoux, Cedric de Bazelaire, Marcela Albiter, Sylvie Giacchetti, Caroline Cuvier, Anne Janin, Marc Espié, Hugues de Thé, Philippe Bertheau

**Affiliations:** 1 AP-HP, Hosp Saint-Louis, Laboratory of Biochemistry, 1 av C. Vellefaux, Paris, 75010, France; 2 CNRS UMR7212/INSERMU944, IUH, 1 av C. Vellefaux, Paris, 75010, France; 3 University Paris Diderot, Sorbonne Paris Cité, Paris, 75010, France; 4 AP-HP, Hosp Saint-Louis, Breast Diseases Center, 1 av C. Vellefaux, Paris, 75010, France; 5 AP-HP, Hosp Saint-Louis, Biostatistic Department, 1 av C. Vellefaux, Paris 75010, France; 6 AP-HP, Hosp Saint-Louis, Laboratory of Pathology, 1 av C. Vellefaux, Paris, 75010, France; 7 INSERM UMR_S728, 1 av C. Vellefaux, Paris, 75010, France; 8 AP-HP, Hosp Saint-Louis, Department of Surgery, 1 av C. Vellefaux, Paris, 75010, France; 9 AP-HP, Hosp Saint-Louis, Department of Radiology, 1 av C. Vellefaux, Paris, 75010, France

**Keywords:** cancer, breast carcinoma, molecular apocrine, estrogen receptor, HER2, GCDFP15, triple negative, basal-like

## Abstract

**Introduction:**

Molecular apocrine (MA) tumors are estrogen receptor (ER) negative breast cancers characterized by androgen receptor (AR) expression. We analyzed a group of 58 transcriptionally defined MA tumors and proposed a new tool to identify these tumors.

**Methods:**

We performed quantitative reverse transcription PCR (qRT-PCR) for *ESR1*, *AR*, *FOXA1 *and *AR*-related genes, and immunohistochemistry (IHC) for ER, PR, Human Epidermal Growth Factor Receptor 2 (HER2), CK5/6, CK17, EGFR, Ki67, AR, FOXA1 and GCDFP15 and we analyzed clinical features.

**Results:**

MA tumors were all characterized by *ESR1*(-) *AR*(+) *FOXA1*(+) and *AR*-related genes positive mRNA profile. IHC staining on these tumors showed 93% ER(-), only 58% AR(+) and 90% FOXA1(+). 67% and 57% MA tumors were HER2(3+) and GCDFP15(+), respectively. Almost all MA tumors (94%) had the IHC signature HER2(3+) or GCDFP15(+) but none of the 13 control basal-like (BL) tumors did. Clinically, MA tumors were rather aggressive, with poor prognostic factors.

**Conclusion:**

MA tumors could be better defined by their qRT-PCR-AR profile than by AR IHC. In addition, we found that HER2 or GCDFP15 protein overexpression is a sensitive and specific tool to differentiate MA from BL in the context of ER negative tumors. A composite molecular and IHC signature could, therefore, help to identify MA tumors in daily practice.

## Introduction

Breast cancer is the most common invasive cancer in women. Sex steroid hormones estrogen and progesterone are key drivers in the carcinogenesis through their actions on estrogen receptor alpha (ER) and progesterone receptor (PR). In daily practice, breast cancer molecular classification is based on the immunohistochemical expression of these receptors (ER and PR) and of Human Epidermal Growth Factor Receptor 2 (HER2), a member of the epidermal growth factor receptor family. However, the androgen receptor (AR), another member of the steroid receptor family, is also largely expressed in more than 70% of breast cancers and is now clearly implicated in the pathogenesis of breast cancer [1]. Although largely co-expressed with ER, AR can also be overexpressed in ER(-) breast tumors [2]. The ER(-) tumors represent 30% of breast cancers and are highly heterogeneous, including at least basal-like (BL) tumors and part of the HER2 positive tumors. Yet, among these ER(-) tumors, several teams have identified the molecular apocrine breast cancer (MA) subtype, characterized by AR expression and AR pathway activation on genome-wide expression analyses, paradoxical expression of genes known to be ER targets or expressed in ER(+) tumors and HER2 overexpression in around 50% of cases [3][4]. The existence of this MA subgroup suggests a new molecular classification for breast cancers, including luminal, MA and BL breast cancer subgroups [5]. AR overexpression may provide a new therapeutic target for breast cancer [6], especially in patients with ER(-) tumors that do not benefit from endocrine or HER2 targeted therapies. A potential therapeutic effect of AR inhibition in MA subtype has already been shown using *in vitro *models [4]. However, there is no clear consensus yet to define the MA subgroup, except by transcriptomic analysis. An easy and reproducible method to identify MA breast cancers is needed to better understand the behavior of these tumors and to enable their inclusion in specific trials.

Here, we used a molecular apocrine qRT-PCR signature initially defined on a set of breast cancer samples annotated with their transcriptional profiles. We retrospectively identified a group of MA tumors based on this signature. We described their clinical, molecular and pathological features and we identified a new simplified immunohistochemical and molecular signature leading to an easy to use and reproducible diagnostic tool for these tumors.

## Materials and methods

### Patients

In order to identify patients with molecular apocrine tumors, we proposed a qRT-PCR molecular apocrine (MA) signature defined by the absence of *ESR1 *overexpression (ER-), *AR *and *FOXA1 *overexpression, as well as overexpression of three of five genes related to the AR pathway (*Agr2, ALCAM, SPDEF, TTF3, UGT2B28A*), according to what was previously described in the literature [4,5].

To validate this MA signature in the context of ER-negative tumors, we constituted a validation set of 45 ER-negative samples with available microarray data (E-MTAB-365, GSE26639) predicted to be molecular apocrine (32 cases) or basal-like (13 cases) by our previously published predictor [7]. These validation data are available in Additional file 1. The qRT-PCR signature discriminated correctly the 32 tumors predicted to be molecular apocrine by the microarray predictor.

With this validated qRT-PCR signature, we retrospectively screened 502 breast cancer patients treated in St. Louis Hospital (Paris) between 1996 and 2008 and have frozen samples available for molecular analysis. We identified 58 molecular apocrine tumors and used 13 basal-like tumors as a control group. Clinical data including age, type of surgery, type of treatment, occurrence and type of relapse and current status were obtained from the Breast Disease Center of the hospital by review of medical charts. Pathological data including histological type, grade, tumor size, peritumoral vascular invasion and stage were recorded. Samples were provided by the biological resource center after approval of the Saint Louis hospital ethical review board (Paris, France: agreement n° DC 2009-929), following the Ethics and Legal national French rules for the patients' information and consent (ANAES, HAS and INCa). All patients were informed of the study and did not oppose it, according to our Institutional Review Board recommendations.

### Molecular analyses

Total RNA was extracted from fresh tumor tissue sections using phenol/chloroform extraction. After a reverse transcription step (Superscript II reverse transcriptase, Life Technologies SAS, Saint Aubin, France), we analyzed *ESR1*, *AR*, *FOXA1 *and *AR*-related genes (*UGT2B28A, ALCAM, AGR2, SPDEF *and *TTF3*) expression using specific primers and probes or Gene Expression Assays (Applied Biosystems Inc., Foster City, CA, USA) (ER [8], AR (Hs00171172_m1), *FOXA1 *(Hs00270129_m1), *UGT2B28A *(Hs00852540_s1), *ALCAM *(Hs00233455_m1), *AGR2 *(Hs00180702_m1), *SPDEF *(Hs01026048_m1), *TFF3 *(Hs00173625_m1) by quantitative real time PCR (qPCR, Taqman 7500, Applied Biosystems Inc.). We performed relative quantification using the TATA box binding protein (*TBP*) gene as the endogenous control and final results were expressed as normalized ratios (target gene/reference gene). *AR *and *FOXA1 *were considered as overexpressed with a relative ratio cut-off at 100. The cut-off ratios were determined on the histogram of mRNA ratio distribution on our tumors. We showed a clear bimodal distribution in all our samples, using the mixture model of two Gaussian distributions with optimal cut-off at the intersection of the probability density functions [9]. We determined *ERBB2 *(HER2) expression level by qRT-PCR as previously described [10]. We also analyzed *TP53 *status by functional assay in yeast (FASAY ) as originally described in [11,12] and as described in [13].

After DNA extraction using phenol/chloroform method, we analysed *PIK3CA *mutational status (hot spots: E542K, E545K and H1047R) by allelic discrimination on a LC480 cycler (Roche Diagnostics, Meylan, France) [14].

### Immunohistochemistry

We performed the following immunohistochemical stainings: ER (clone 6F11, dilution 1/50, Novocastra Laboratories, Neucastle Upon Tyne, UK), PR (clone 312, dilution 1/75, Novocastra), HER2 (clone CB11, dilution 1/250, Novocastra), Ki-67 (clone Mib 1, dilution 1/100, Dako-France Les Ulis, France), CK5/6 (clone D5/16 B4, dilution 1/50, Dako), CK17 (clone E3, dilution 1/75, Dako), EGFR (clone Dak-H1-WT, dilution 1/20, Dako), AR (clone AR441, dilution 1/20, Dako), FOXA1 (clone 2F83, dilution 1/500, Abcam, paris, France) and GCDFP15 (clone 23A3, dilution 1/50, Dako). Evaluation of HER2 immunostaining was scored according to ASCO guidelines [15,16]. Dual silver *in situ *hybridization (Roche Ventana, Tucson Arizona, USA) was used in 4 HER2(2+) cases and the polyclonal anti-HER2 antibody (Dako) was used in another HER2(2+) case. Cytoplasmic immunostainings for GCDFP15 and for basal cytokeratins were considered positive if at least 5% of the tumor cells were positive. All other immunostainings were nuclear stainings (ER, PR, FOXA1, AR) and were considered positive if at least 10% of the tumor cells were positive. For Ki67 immunostaining, the percentage of positive nuclei was noted.

### Statistical analyses

Immunohistochemical protein and mRNA expression were compared between the two tumor groups using Fisher's exact tests. Performance of immunohistochemical signatures to discriminate between apocrine and basal-like tumors was expressed in terms of sensitivity, specificity and the area under the receiver operating characteristics curve (AUC). The survival data, overall survival (OS) and disease free survival (DFS), were estimated using Kaplan-Meier product-limit estimator.

A tumor signature was developed by using classification trees (CART algorithm) [17]. Briefly, this method builds up a tree model to reduce misclassification rates or tree deviance, with a penalization for the tree complexity. The CART model was first built up to a maximum tree, then upward tree pruning through 10-fold cross-validation was used to cut the tree down [18]. Multiple correspondence analysis (MCA) was used to graphically display the association between the expressions of the different proteins. All tests were two-sided and *P*-values <0.05 were considered as indicating significant association. Analyses were performed using the R statistical software version 2.10.1 (The R Foundation for Statistical Computing, Vienna, Austria) [19], with the package tree for CART analyses [20].

## Results

### Clinical description of the group of patients

We constituted a cohort of 58 breast cancers presenting the qRT-PCR signature of the molecular apocrine subgroup and we used the 13 basal-like tumors as a control group. The molecular apocrine group presented a mean age at diagnosis of 54 years and a majority of patients had a clinical tumor size larger than 2 cm (T2-T3-T4, 81%). All but seven patients received chemotherapy (neoadjuvant 15%, adjuvant 59%, neoadjuvant and adjuvant 14%) and 28% received trastuzumab (Table 1). Clinical data for basal-like control tumors are also available in Table 1.

**Table 1:** Patient and tumor characteristics

Characteristics	Molecular apocrine No. patients (%)	Basal-like No. patients (%)
**All patients**	58	13
**Age at diagnosis (range) **	54 y.o. (32 to 86)	43 y.o. (27 to 63)
**Menopausal status** ** - premenopausal** ** - postmenopausal**	26 (45) 32 (55)	10 (77) 3 (23)
**Clinical tumor size** ** -T0-T1** ** -T2-T3-T4**	11 (19) 47 (81)	0 (0) 13 (100)
**Histology** ** - Invasive ductal** ** with Paget disease** ** with apocrine differentiation** ** with squamous metaplastic areas**	58 (100) 4 (7) 4 (7) 1 (2)	13 (100) 1 (8)
***In situ *component**	44 (76)	3 (23)
**Pathological nodal involvment** ** -N0** ** -N1, N2, N3** ** no axillary dissection **	22 (38) 33 (57) 3 (5)	11 (85) 2 (15)
**Grading SBR** ** -G2** ** -G3 **	15 (26) 43 (74)	1 (8) 12 (92)
**Lymphovascular invasion**	28 (48)	1 (8)
**Treatments:** **Surgery** ** - lumpectomy** ** - mastectomy** ** - no** **Chemotherapy (CT)** ** - neoadjuvant CT** ** - adjuvant CT** ** - neoadjuvant and adjuvant CT** **- no** **Radiotherapy** **Trastuzumab **	18 (31) 39 (67) 1 (2) 9 (15) 34 (59) 8 (14) 7 (12) 41 (71) 16 (28)	0 (0) 13 (100) 0 (0) 0 (0) 1 (8) 12 (92) 0 (0) 10 (77) 0 (0)

### Molecular description of the two groups

We analyzed the two groups for *ESR1*, *ERBB2*, *AR*, *FOXA1 *and five *AR*-related genes expression by qRT-PCR. Molecular apocrine tumors showed lack of *ESR1 *expression (ER-), overexpression of *AR*, *FOXA1 *(Table 2) and of three out of five *AR*-related genes (data not shown). *ERBB2 *expression level was positive in 68% of cases. Conversely, all BL tumors showed lack of *ESR1*, *FOXA1 *and *ERBB2 *expression and also lack of *AR *in 11/13 cases (85%) (Table 2).

**Table 2:** Molecular characteristics of the MA and BL tumors groups (%)

	Molecular apocrine	Basal-like	*P*-value
**No. patients**	58	13	
**ER expression (mRNA) (pos >20)** **-negative **	58 (100)	13 (100)	>0.99
**HER2 expression (mRNA) (pos >7)** **-positive** **-negative** **-nd**	38 (68) 18 (32) 2	0 (0) 13 (100) 0	<0.0001
**AR expression (mRNA) (pos >100)** **-positive** **-negative**	58 (100) 0 (0)	2 (15) 11 (85)	<0.0001
**FOXA1 expression (mRNA) (pos >100)** **-positive** **-negative**	58 (100) 0 (0)	0 (0) 13 (100)	<0.0001
***PIK3CA *status:** **-wild type** **-mutated** **- exon 9: E542K and E545K** **- exon 20: H1047R**	47 (81) 11 (19) 1 10	13 (100) 0 0 0	0.20
**TP53 status (FASAY test)** **-functional** **-nonfunctional**	29 (50) 29 (50)	0 (0) 13 (100)	0.0005

AR, androgen receptor; BL, basal-like; ER, estrogen receptor; HER2, Human Epidermal Growth Factor Receptor 2; MA, molecular apocrine.

We assessed mutations in the three hotspots of the *PIK3CA *gene (E542K, E545K and H1047R) in both tumor groups. In the MA group, 11/58 (19%) mutations on *PIK3CA *could be detected compared to no mutation case in the BL group (Table 2) with a majority of H1047R mutations, located in the catalytic domain of PIK3CA (exon 20). However, the difference between the two groups was not statistically significant.

Additionally, we analyzed the *TP53 *functional status by the FASAY assay and 29/58 (50%) of MA tumors were p53 nonfunctional compared to 100% of the BL tumors (Table 2).

### Pathological and immunohistochemical description of the two groups

As shown in Table 1, all MA tumors were ductal invasive carcinoma, with morphological apocrine differentiation in four tumors (7%), Paget's disease in four others (7%) and *in situ *component in 44 patients (76%). Tumors were grade 3 in 43 patients (74%), grade 2 in 15 patients (26%), and lymphovascular invasions were observed in 28 cases (48%). Pathological data for the BL tumors are also available in Table 1.

**Figure 1:**
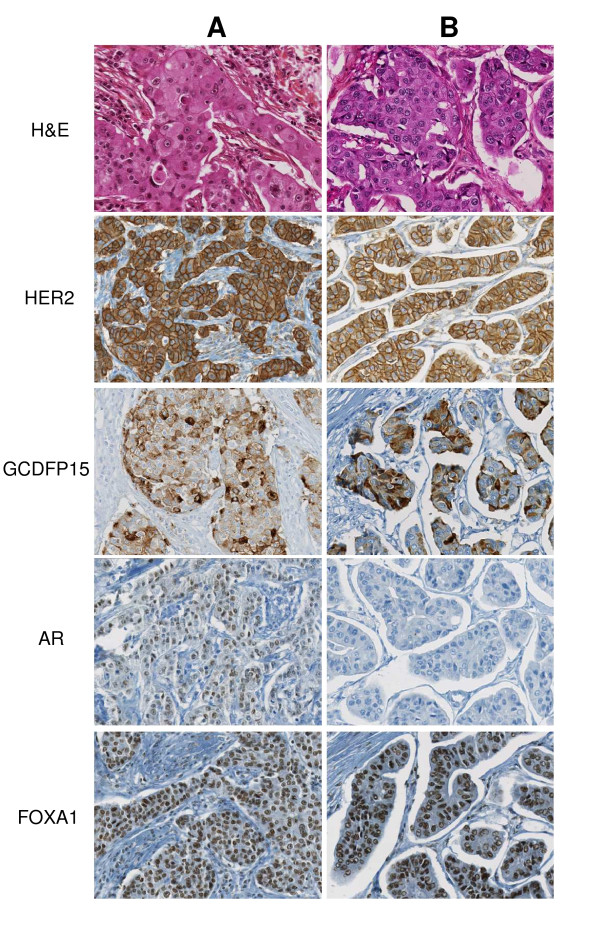
**Histopathological features of two molecular apocrine cases**. Both cases show apocrine differentiation on H&E stainings, as well as positive immunostainings for HER2, GCDFP15 and FOXA1. AR immunostaining is positive in case **A **but negative in case **B**. AR, androgen receptor; HER2, Human Epidermal Growth Factor Receptor 2.

IHC profiles among the MA tumors are shown in Table 3 and two examples are given in Figure 1. Almost all tumors were ER(-), PR(-) and 67% were HER2 (+). Among five HER2(2+) cases, four were *ERBB2 *amplified as shown by Silver *In Situ *Hybridization and one case was found negative with a second anti-HER2 antibody (polyclonal, Dako). FOXA1 and AR immunostainings were positive in 90% and 58% of MA tumors, respectively, and 57% were positive for GCDFP15 with 5% to 90% stained tumor cells (median 50%). Conversely, the majority of cases were EGFR(-), CK5/6(-), and CK17(-) and 10/55 (18%) tumors were below 20% Ki67 staining (median 30% stained cells).

**Table 3:** Immunohistochemical description of the MA and BL tumors groups (%).

	Molecular apocrine	Basal-like	*P*-value
**No. patients**	58	13	
**ER(-)**	54 (93)	13 (100)	>0.99
**PR(-)**	56 (97)	13 (100)	>0.99
**HER2(3+)**	39 (67)	0 (0)	<0.0001
**GCDFP15(+)**	33 (57)	0 (0)	<0.0001
**AR(+)**	33 (58)	0 (0)	<0.0001
**FOXA1(+)**	52 (90)	4 (31)	<0.0001
**EGFR(-)**	40 (70)	4 (31)	0.012
**CK5/6(-)**	51 (89)	5 (38)	0.0003
**CK17(-)**	54 (95)	5 (38)	<0.0001
**Ki67 median (range of % stained cells)**	30 (0 to 80)	50 (0 to 90)	0.27

AR, androgen receptor; BL, basal-like; ER, estrogen receptor; HER2, Human Epidermal Growth Factor Receptor 2; MA, molecular apocrine; PR, progesterone receptor.

Among the four tumors with morphological apocrine differentiation, all were ER(-) and GCDFP15(+), two were HER2(3+), two were AR(+) and three were EGFR(+).

In comparison, all the 13 BL tumors were ER(-) PR(-) HER2(-), AR(-) and GCDFP15(-). Thirty-one percent of BL tumors were FOXA1(+), 69% EGFR(+) and 62% were CK5/6(+) and CK17(+). Two BL tumors (15.4%) were below the 20% threshold for Ki67 staining (median 50% stained cells). There was no difference between the two groups for Ki67 expression (Table 3).

### Correlation of molecular and immunohistochemical markers of the two groups

We compared the two groups of tumors for *AR *and *FOXA1 *expressions at protein and mRNA levels. As mentioned, all the MA tumors overexpressed *AR *and *FOXA1 *mRNA but only 58% and 90% were positive for AR and FOXA1 with IHC, respectively.

**Figure 2:**
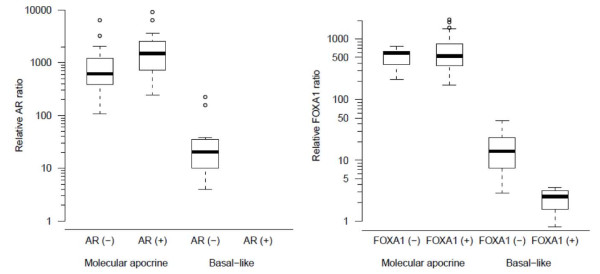
**Relationship between AR and FOXA1 qRT-PCR and IHC in the MA and BL tumors groups**. Positive cut-off ratio is 100. Boxplots indicate first quartile, median and third quartile. Outliers are indicated by small circles. AR, androgen receptor; BL, basal-like; IHC, immunohistochemistry; MA, molecular apocrine.

The relative mRNA *AR *ratio was strongly positive in all MA cases (Figure 2), with a higher ratio in AR IHC(+) group compared to AR IHC(-) tumors (mean ratio of 2,207 and 1,062, respectively). In the BL subgroup of tumors, we observed a good correlation between AR protein and mRNA levels, all cases showing weak or negative expression (mean value 45.4). These data suggest that *AR *mRNA determination is more sensitive than AR IHC in MA tumors.

The relative *FOXA1 *ratio was also positive in all MA cases whatever the IHC staining (Figure 2), but the mean *FOXA1 *ratio was not significantly different in the IHC(+) and IHC (-) group (mean ratio 660.3 versus 524.3) In the BL subgroup, lack of FOXA1 expression was concordant at the mRNA and protein levels but four FOXA1 IHC(+) tumors could not be detected at the mRNA level.

These results suggest that *AR *and *FOXA1 *mRNA signatures could not be transposed directly into protein levels and that AR IHC and FOXA1 IHC do not correctly identify the MA subgroup.

### Definition of an immunohistochemical and molecular signature of the MA subgroup of tumors

As shown in Table 4, AR IHC positivity, evaluated alone in a ER(-) setting, had a good specificity (100% (95% CI: 75 to 99)) for MA tumors but a poor sensitivity (58% (95% CI: 44 to 72)). Combining AR IHC with FOXA1 IHC did not improve the specificity and the sensitivity. Indeed, the IHC signature ER(-) AR(+) FOXA1(+), although being the IHC counterpart of the molecular definition, was found in only 57% of MA tumors.

**Table 4:** Sensitivity and specificity of the IHC signatures of MA tumors in ER(-) subgroup.

IHC biomarker	Sensitivity (95% CI)	Specificity (95% CI)	AUC
**AR(+)**	58% (44 to 72)	100% (75 to 99)	0.792 (0.726 to 0.859)
**AR(+) and FOXA1(+)**	57% (43 to 71)	100% (75 to 99)	0.787 (0.721 to 0.853)
**HER2(3+) or GCDFP15(+)**	94% (85 to 99)	100% (75 to 99)	0.972 (0.942 to 0.999)

AR, androgen receptor; ER, estrogen receptor; HER2, Human Epidermal Growth Factor Receptor 2; IHC, immunohistochemistry; MA, molecular apocrine.

**Figure 3:**
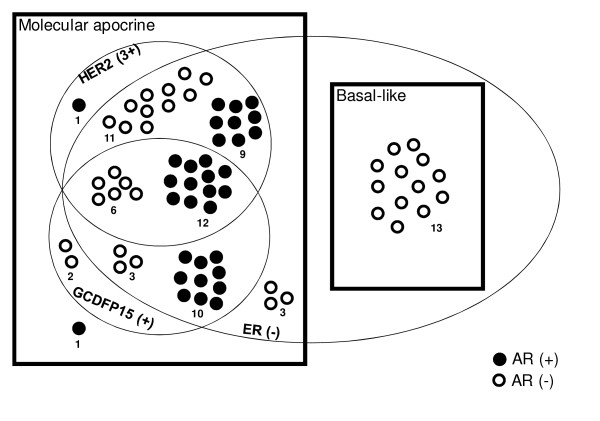
**Distribution of the MA and BL tumors according to ER, HER2, GCDFP15 and AR immunostainings**. Numbers of tumors in each subgroup are indicated. AR, androgen receptor; BL, basal-like; ER, estrogen receptor; HER2, Human Epidermal Growth Factor Receptor 2; MA, molecular apocrine.

**Figure 4:**
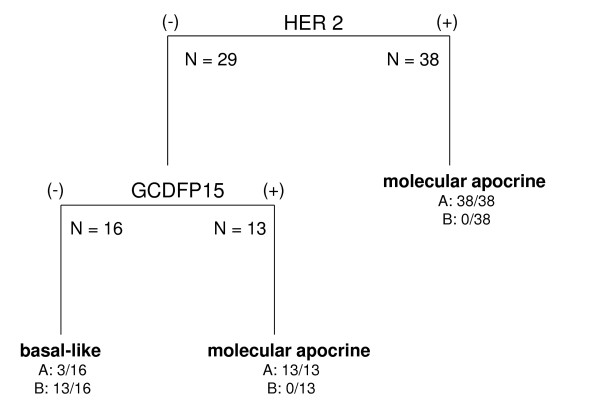
**Classification tree in the 67 ER(-) patients**. Bold basal-like and molecular apocrine labels refer to the classification result at the terminal nodes of the tree (leafs). **A **and **B **denote the numbers of MA and BL tumor patients over the total number of patients at the nodes. BL, basal-like; ER, estrogen receptor; MA, molecular apocrine.

We then studied how the 58 MA and 13 BL tumors were distributed according to the following IHC markers: ER, HER2, GCDFP15 and AR (Figure 3). It appeared on this figure that "HER2(3+) OR GCDFP15(+)" cases accounted for 51 out of 54 ER(-) tumors (94%) but was never observed in BL tumors. This IHC signature "HER2(3+) OR GCDFP15(+)" had a sensitivity and a specificity for apocrine tumors of 94% and 100%, respectively (Table 4) and is also shown in a tree diagram displaying the best possible marker combination to discriminate MA and BL tumors in the ER negative context (Figure 4). Combining *AR *gene expression pathway and this IHC signature seems finally to be an efficient composite approach to define MA tumors.

### Survival analysis of the two groups

**Figure 5:**
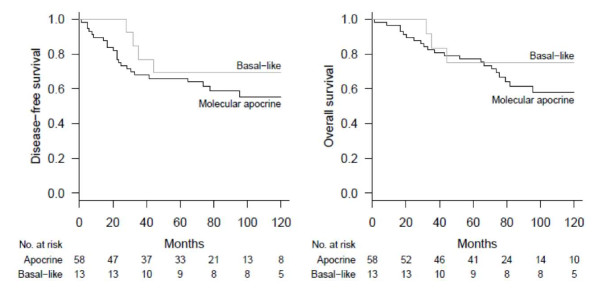
**DFS and OS curves of the 58 MA and 13 BL tumors**. DFS, disease free survival; OS, overall survival.

In the MA subgroup, after a median follow-up of 94 months (range: 0.8 to 175 months), 33 events (local recurrence *n *= 8, contralateral *n *= 3, distant metastasis *n *= 22), and 21 deaths occurred. Five-year disease free survival (DFS) and overall survival (OS) were 66% and 77%, respectively. In the BL subgroup, the median follow-up was 122 months (range: 28; 195) and five-year DFS and OS were 79% and 73%, respectively (Figure 5). No statistical difference was observed between the two groups (*p *= 0.35 for DFS and *p *= 0.22 for OS). Also, in the MA subgroup, there was no statistically significant difference in terms of DFS according to the IHC status AR(+)/AR(-), HER2(3+)/HER2(-) or GCDFP15(+)/GCDFP15(-) of the tumors (see Additional file 2).

## Discussion

Breast cancers are highly hormone-dependent tumors. The prominent role of the estrogen and progesterone receptors as prognostic factors and treatment targets is well established. Despite the fact that androgen expression in breast cancer has been known for a long time, interest for this type of hormone nuclear receptor as a possible biomarker is more recent. The description of the MA breast cancer subgroup raises new questions about AR implication in prognosis or prediction of response to hormone blockade in an ER-negative setting. Moreover, easy and robust identification of this new tumor subgroup is needed for proper evaluation. We report here a series of 58 MA breast tumors and compare protein and mRNA expression of genes included in the transcriptomic MA signature [4]. We propose a sensitive and specific composite immunohistochemical and molecular diagnostic signature.

MA tumors were originally described by Farmer *et al. *[3] who identified, with microarray data, a new subgroup of breast tumors which often displayed apocrine morphology (five out of six cases), and they, therefore, suggested that these tumors be called "MA breast carcinomas". To date, data have been somewhat confusing regarding the relationship between MA breast tumors and pathologically-defined apocrine tumors. On breast tissue sections, histological apocrine changes are defined by eosinophilic cytoplasms with fine granularity, large nuclei with occasional large nucleoli and frequent apical snouts [21,22]. The term "apocrine carcinoma", as defined by microscopical examination, should be used for neoplasms composed of at least 90% apocrine type epithelium [23]. These tumors account for 0.5% to 4% of breast carcinomas and mostly occur at advanced ages. Their prognosis is similar to that of other breast carcinomas [24]. In our study, we found only four morphological apocrine tumors among 58 MA cases, strongly suggesting that the MA subtype could in fact be much broader than initially reported by Farmer *et al. *[3].

A frequently reported feature of apocrine differentiation, whatever being defined morphologically or molecularly, is a strong androgen receptor expression in an otherwise ER(-) background. Indeed, although sometimes reported to belong to several molecular subtypes of breast cancer [25], morphological apocrine carcinomas are almost always ER(-) tumors [26] and, among 19 cases, all were ER(-), PR(-) and strongly AR(+) by IHC [27]. Currently, the MA subgroup is characterized by *AR *gene overexpression and Niemeier *et al. *[28] suggested that the ER(-)/PR(-)/HER2(+)/AR(+) or ER(-)/PR(-)/HER2(-)/AR(+) IHC signature could include the "MA group" described previously by Farmer *et al. *[3]. However, our results raise the question whether AR, as assessed by IHC, is a good marker to define this tumor subgroup. Indeed, although this marker was pretty specific for MA carcinomas, since it was absent in BL cancers, we found that only 58% of MA tumors were IHC AR(+), thus making this marker not sensitive enough for clinical use. Conversely, *AR *mRNA, evaluated by qRT-PCR, was highly expressed, whatever the level of IHC expression. Doane *et al. *[4] also found that only 50% of MA tumors expressed AR by IHC, despite high *AR *expression levels in microarrays data. This dissociation between AR mRNA and protein expression may be due to a lower sensitivity of immunohistochemistry or a faster AR degradation due to activation, leading to undetectable protein.

AR related genes *AGR2*, *ALCAM SPDEF *and *TTF3 *were found in the top 50 significantly overexpressed genes in the transcriptomic profiles of MA tumors [4]. Some of them, like *AR *itself or *SPDEF*, have putative androgene response elements (ARE). Moreover, *AGR2 *(androgen-inducible gene anterior gradient-2) is highly induced by androgen in an androgen receptor (AR)-dependent manner. After all, the main trait of MA tumors is the activation of the AR pathway, and AR target genes could help to identify this tumor subgroup.

FOXA1 (Forkhead box protein A1), a member of the forkhead class of DNA-binding proteins, is a pioneer factor facilitating the recruitment of ER and AR to their response elements on the genome. In breast cancer, FOXA1 expression is highly correlated with ER(+), PR(+) and endocrine signaling, and may have a prognostic value in ER(-) tumors [29]. Doane *et al*. reported that FOXA1 is overexpressed in the MA subtype [4]. In our study, according to the transcriptomic definition of our MA group (ER(-) AR(+) FOXA1(+)), up to 90% of MA tumors were also FOXA1 positive by IHC. However, 31% of BL tumors were positive as well, thus making FOXA1 immunostaining not specific enough to identify MA tumors.

GCDFP15, the product of the AR-target gene *PIP *(Prolactin Induced Protein) [30], has been initially described as an apocrine marker. It is present at high concentrations in breast cyst fluid [24] but also expressed in ER(+) or ER(-) breast carcinomas. To date, it is routinely used for the diagnosis of metastasis of unknown origin to identify tumors coming from the breast, with 95% specificity and 74% sensitivity [24,31]. GCDFP15 mRNA are also overexpressed in MA tumors [4]. However, in our study, GCDFP15 was not more sensitive than AR to identify MA tumors when analyzed alone by IHC (57% IHC GCDFP15(+) in the MA group). Our results also showed that GCDFP15 negativity was constant in BL tumors. Therefore, in ER(-) tumors, GCDFP15 positive IHC staining seems to be highly specific of MA cancers but not very sensitive of this subtype.

We then analyzed the sensitivity and specificity of the potentially relevant following IHC signatures: AR alone, AR AND FOXA1, HER2 OR GCDFP15. Doane *et al. *[4] also proposed one IHC signature without AR: ER(-) PR(-) ALCAM(+) and SPDEF(+), but this signature was validated on only 10 MA tumors. Besides that, ALCAM/SPDEF immunostainings are not widely used and have not been standardized so far. Niemeier *et al. *[28] reported that the tumors with the IHC profiles ER(-) HER2(3+) AR(+) or ER(-) HER2(-) AR(+) may be MA tumors but it is likely that other MA tumors will be missed with this definition centered on AR IHC. We propose here, in ER(-) breast tumors, a composite signature including AR gene expression pathway analysis by qRT-PCR in addition to "HER2(3+) or GCDFP15(+)" protein expression by IHC. Our IHC signature is composed of validated immunostainings, with international guidelines, used on a daily basis in pathology laboratories. The qRT-PCR profile can be easily obtained on frozen tissue, provided that a frozen-tissue workflow is organized. The validation of quantification on formalin-fixed paraffin-embedded tissues is under way and will allow qRT-PCR analysis on almost all samples.

We also analyzed other molecular alterations in these MA tumors. The phosphatidylinositol-3 kinase (PI3K) signaling pathway is crucial for cell growth and cell survival. Mutations in the gene encoding the p110a (*PIK3CA*) subunit of PI3K are commonly found in breast cancer. Gonzales-Angulo *et al. *[32] reported a possible association between the level of AR and *PIK3CA *mutations. In our study, although we found *PIK3CA *mutations only in MA tumors, the difference was not statistically significant; however, the number of patients was low. Also, no difference in *AR *mRNA expression level could either be detected in the *PIK3CA *mutated group of MA tumors compared to the *PIK3CA *wild type counterpart (data not shown).

*TP53 *mutations are commonly found in ER(-) breast cancer, and nearly 90% in the triple negative subgroup, but were present in only 50% of our MA tumors. In prostate cancer cells, it was described that loss of p53 function contributes to increased AR expression [33]. In our study, no differences in AR expression could be detected in the p53 nonfunctional subgroup of MA tumors compared to the functional p53 group (data not shown). This potential link between AR and p53 should be further evaluated.

In breast cancers, AR is overexpressed in up to 70% of cases [34], mainly in luminal and low grade tumors [6,35]. This expression has been associated with a favorable outcome in ER(+) tumors [36,37] as well as in ER(-) tumors [35,38,39]

Little is known about the clinical outcome of MA carcinomas, as very few tumors were available in previous transcriptomic studies (six specimens in the works of Farmer *et al. *[3], and nine in Doane *et al. *[4]). In our study cohort, demographic or clinical presentation did not show specific features. We did not find any association with Cowden disease, as reported by some authors [40]. Though limited by small sample size and retrospective review, we found that MA tumor phenotypes appeared to be rather aggressive, with a high proportion of poor prognosis factors (grade SBR3, lymphovascular invasion, node involvement). They were also associated with a poor clinical outcome despite the fact that the wide majority of patients received chemotherapy.

Recently, Lehmann *et al. *[41] re-analyzed 21 breast data sets, including 587 triple negative breast carcinomas (TNBC), and identified a new TNBC subtype called "luminal androgen receptor" (LAR) type. Though not named "molecular apocrine" and all being HER2(-), these 62 LAR tumors seemed to be included in the MA group. Relapse free survival was significantly decreased in the LAR subtype compared with other TNBC subtypes.

## Conclusions

Finally, our study demonstrates that we could accurately identify MA breast tumors by AR pathway analysis by qRT-PCR, in addition to HER2 or GCDFP15 protein overexpression by IHC. This composite tool may be sensitive, specific and useful to differentiate ER negative tumors in MA or BL, in daily practice. Accurate identification of MA tumors could help to include these patients with rather aggressive tumors in specific "AR-pathway" therapeutic trials or to identify other therapeutic targets.

## Abbreviations

AGR2: androgen-inducible gene anterior gradient-2; AR: androgen receptor; ARE: androgene response element; AUC: area under the receiver operating characteristics curve; BL: basal-like; DFS: disease free survival; ER: estrogen receptor alpha; FASAY: functional assay in yeast; HER2: Human Epidermal Growth Factor Receptor 2; IHC: immunohistochemistry; LAR: luminal androgen receptor; MA: molecular apocrine; MCA: multiple correspondence analysis; OS: overall survival; PI3K: phosphatidylinositol-3 kinase; q-RT PCR: quantitative reverse transcription PCR; TNBC: triple negative breast cancer

## Competing interests

The authors declare that they have no competing interests.

## Authors' contributions

JLC, ASH and PB designed the study, collected and analyzed the data, and wrote the manuscript. JLC and ASH contributed equally to this work. RP carried out all the statistical analyses. MB carried out the biological data collection, the data management and contributed to data analysis. FB carried out all immunohistochemical stainings.

HH and SLD carried out the clinical data collection with review of the medical charts and survival follow-up. PB and AdR carried out all diagnoses and pathological interpretations. LCD, EB, CdB and MA obtained fresh frozen tissues sections under optimal conditions for all the biological studies. SG and CC enrolled patients. ME contributed to the design of the study, enrolled patients and contributed to data collection. PdC, AJ and HdT read and critically revised the manuscript. All authors read and approved the final manuscript.

## Supplementary Material

Additional file 1 **Molecular apocrine qRT-PCR signature in the 45 ER(-) tumors defined by the microarray predictor**. The 45 tumors are defined to be molecular apocrine (MA) or basal-like (BL) by the microarray predictor. The mRNA expression of *AR, FOXA1*, *AGR2, ALCAM, SPDEF*, *UGT2B28A *and *TTF3 *is evaluated by q RT-PCR, expressed as relative ratio and compared between transcriptionaly defined MA and BL tumors. Click here for file

Additional file 2 **DFS curves of the 54 ER(-) MA tumors according to AR, HER2 and GCDFP15 immunostainings**. Click here for file
