# Supplementary figures and images for: Molecular apocrine breast cancers are aggressive estrogen receptor negative tumors overexpressing either HER2 or GCDFP15
Source: Breast Cancer Res. 2013 May 11;15(3):R37. doi: 10.1186/bcr3421 (PMC4053236; doi:10.1186/bcr3421)

## Slide 1
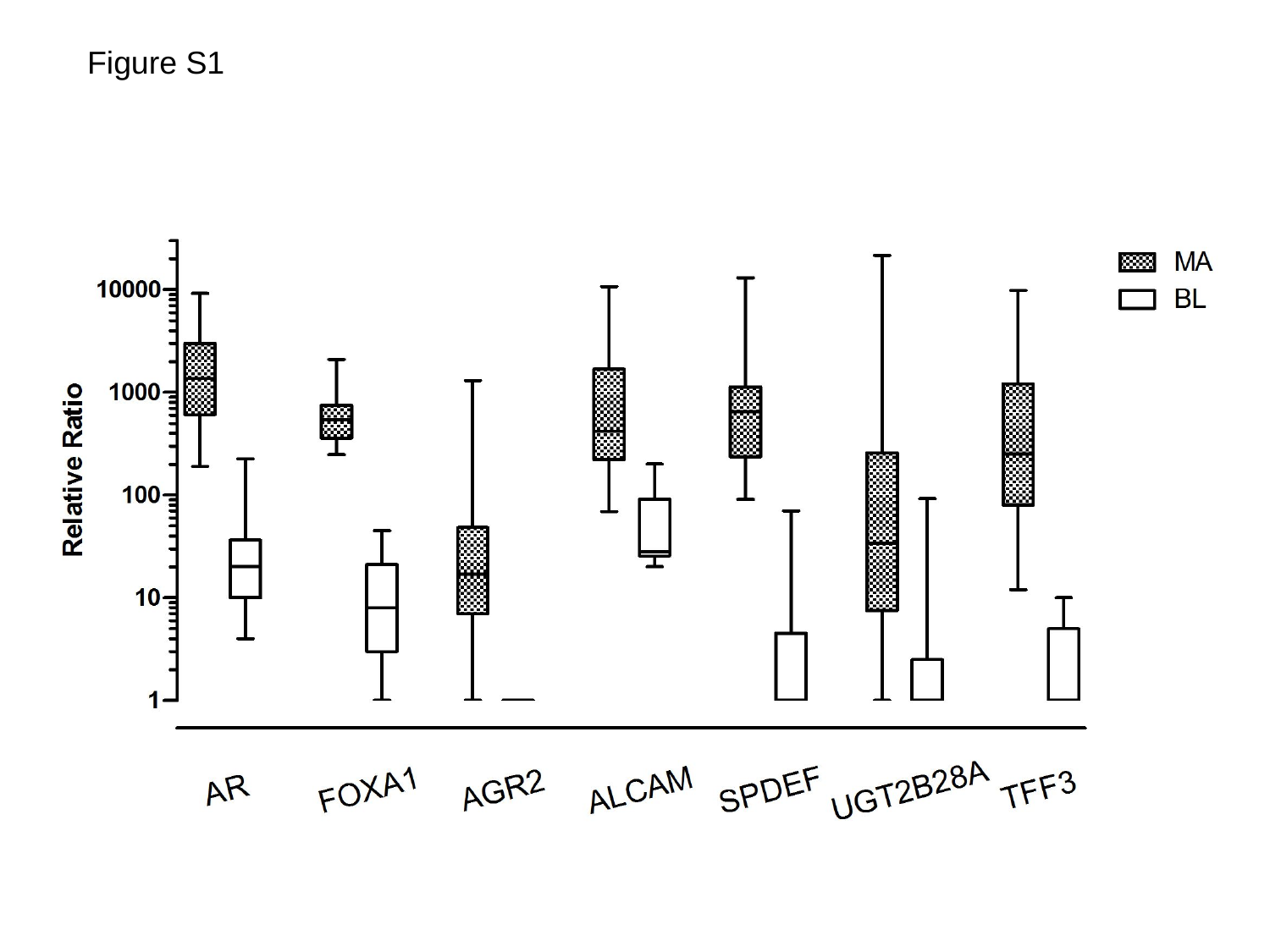

Figure S1

Supplement: Additional file 1 — Molecular apocrine qRT-PCR signature in the 45 ER(-) tumors defined by the microarray predictor. The 45 tumors are defined to be molecular apocrine (MA) or basal-like (BL) by the microarray predictor. The mRNA expression of AR, FOXA1, AGR2, ALCAM, SPDEF, UGT2B28A and TTF3 is evaluated by q RT-PCR, expressed as relative ratio and compared between transcriptionaly defined MA and BL tumors. [file bcr3421-S1.PPT]

## Slide 1
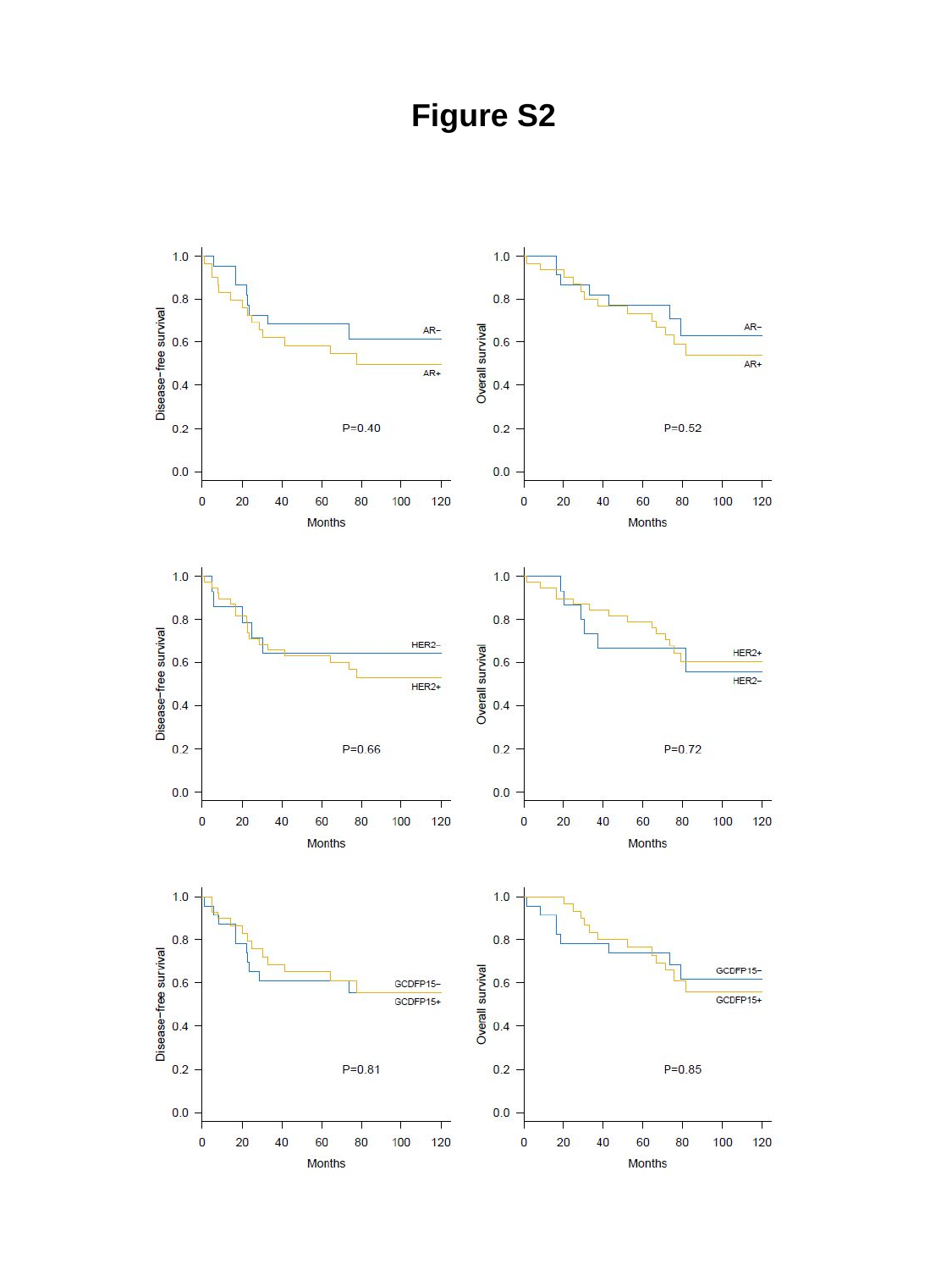

Figure S2

Supplement: Additional file 2 — DFS curves of the 54 ER(-) MA tumors according to AR, HER2 and GCDFP15 immunostainings. [file bcr3421-S2.PPT]
